# Selenium deficiency and scurvy due to an imbalanced diet of snacks and lacto-fermenting drinks: a case report of a 7-year-old boy with autism spectrum disorder

**DOI:** 10.1186/s40795-023-00703-2

**Published:** 2023-03-08

**Authors:** Makoto Okada, Yugo Nagayama, Hitomi Saiki, Kazutoshi Ito, Shuichi Yatsuga, Shinichiro Nagamitsu

**Affiliations:** Department of Pediatrics, Faculty of Medicine, Fukuoka University, 7-45-1 Nanakuma, Jonan-ku, Fukuoka, 814-0180 Japan

**Keywords:** Autism spectrum disorder, Selenium deficiency, Scurvy

## Abstract

**Background:**

There have been reports of isolated trace elements or vitamin deficiencies due to imbalanced diets, but no cases of selenium deficiency combined with scurvy have been reported.

**Case presentation:**

A 7 year-old boy diagnosed with autistic spectrum disorder and mild psychomotor retardation, started an imbalanced diet including specific snacks and lacto-fermenting drinks from 5 years of age. Gingival hemorrhage and perioral erosions occurred at 6 years and 8 months of age, and he was referred to our hospital at 7 years of age. Slight tachycardia was found. Serum vitamin C level was 1.1 µg/dL (reference range (rr): 5–17.5 µg/dL), and selenium level was 2.8 µg/dL (rr: 7.7–14.8 µg/dL). He was diagnosed with both selenium deficiency and scurvy. Multivitamins and sodium selenate were administered for 12 days during admission, and symptoms of selenium deficiency and scurvy improved. After discharge, symptoms abated following the administration of multivitamins and regular administration of sodium selenate every 3 months.

**Conclusions:**

We report a complicated case of both selenium deficiency and scurvy due to an imbalanced diet of snacks and lacto-fermenting drinks in a 7-year-old boy with autism spectrum disorder. In patients with imbalanced diet, regular blood tests including trace elements and vitamins are necessary.

## Background

Selenium has many biological effects, such as the regulation of the arachidonic acid cascade and the enhancement of apoA-I expression through its effects on PPARγ and NF-κB. Selenium deficiency is associated with enhanced inflammation and improves the expression of selenoproteins, which are involved in antioxidative defense reactions [1]. Selenium deficiency is induced following long-term enteral or intravenous nutrition without selenium, dialysis, and eating disorders [2–5]. There are various symptoms of selenium deficiency, such as whitened nails, hair loss, dermatitis, muscle weakness, cardiac myopathy, and abnormal laboratory findings suggesting macrocyticanemia, liver dysfunction and abnormal thyroid function [6].

Vitamin C is an essential cofactor for collagen biosynthesis, carnitine, catecholamine metabolism, and dietary iron absorption. Vitamin C deficiency induces scurvy characterized by the inability to build up the collagen triple helix structure that makes up the dermis, ligaments, tendons, bones, cartilage, and blood vessels, resulting in vessel wall fragility and failure of bone and tooth development [7]. Symptoms of scurvy vary but generally follow a predictable course, especially bleeding of the skin, gums, and joints [8]. Thioredoxin reductase as a selenoenzyme was reported to function as a cytosolic ascorbyl free radical reductase, complementing intracellular ascorbate recycling by membrane-bound NADH-dependent reductase [9]. Thus, combined selenium and vitamin C deficiency are expected to exacerbate the pathological consequences of dietary vitamin C deficiency.

Autism spectrum disorder (ASD) is a developmental disorder characterized by poor interpersonal relationships and strong dietary preoccupation, which is known to cause vitamin and mineral deficiencies. Although there have been a few reports about scurvy [10] and only selenium deficiency [11] in ASD children, there are no reports of ASD children having a combination of scurvy and selenium deficiency. In this report, we describe a case of an ASD child with scurvy and selenium deficiency due to an imbalanced fixed diet of snacks and lacto-fermenting drinks.

## Case presentation

A 7-year-old boy was referred to our hospital due to gingival bleeding for 4 months. He was born a twin at term by caesarean section with a birth weight of 2.51 kg. There were no prenatal or postnatal complications. His twin brother who was diagnosed with attention deficit hyperactivity disorder (ADHD) had normal eating habits. The Kyoto scale of psychological development test was performed at 1 year and 3 months of age, and he was diagnosed with psychomotor retardation due to a developmental quotient (DQ) score of 51. An imbalanced diet consisting of only bananas and sponge cake was started at 1.5 years of age, and changed to only two 57 g boxes of snacks made primarily from processed fried potatoes and two liters of lacto-fermenting drinks in a day. Gingival hemorrhage and perioral erosions presented at 6 years and 8 months of age. At the local clinic for the common cold, blood tests showed anemia with hemoglobin at 7.0 g/dL and ferric pyrophosphate was initiated. He was finally diagnosed with ASD according to DSM 5 at that time.

He was 110 cm (-2.07 SD) in height, and his weight was 19.05 kg at admission. Vital signs were as follows: body temperature of 36.7 ºC, blood pressure of 90/80 mmHg, resting heart rate of 117 beats per minute, respiratory rate of 28 breaths per minute, and oxygen saturation of 98% on room air. Despite the good general condition, there were dry lips, erosion around the lips, gingival swelling, and gingival hemorrhage. All physical examinations were normal. There were no symptoms of nail whitening, nail deformity, hair loss, discoloration of the hair, myalgia of the lower limbs, weakness of extremities, difficulty walking, subcutaneous bleeding, thigh bleeding, purpura, or petechiae around hair follicles.

Laboratory studies showed AST, 98 IU/L(reference range (rr): 24–38 µg/dL); ALT, 64 IU/L(rr: 9-28 IU/L); CK, 45 IU/L (rr: 46-230 IU/L); TSH, 2.55 µIU/mL (rr: 0.44-4.1 IU/L); FT4, 1.36 ng/dL (rr: 1.03-2.00 ng/dL); vitamin C level, 1.1 µg/dL (rr: 5.0–17.5 µg/dL); selenium level, 2.8 µg /dL (rr: 7.7–14.8 µg /dL ); retinol protein, 1.3 mg/dL (rr: 2.5–7.1 mg/dL); vitamin B1, 22 ng/mL (rr: 24–66 ng/dL); and folic acid, < 1.3 ng/mL (rr: 4.0–13.0 ng/mL). Echocardiograms did not show any pathological Q-wave or ST segment and T-wave changes. A lumbar and head MRI showed no hemorrhagic lesions.

Gingival bleeding was thought to be symptoms of scurvy. Elevated AST/ALT and perioral erosions and tachycardia and lip dryness were thought to be symptoms of selenium deficiency. We started the administration of sodium selenite via intravenous infusion (40 µg/d) and multivitamins including 50 mg/day of vitamin C via intravenous infusion after hospitalization. The meal form innovations were made by the nutritionists to try the intake of oral selenium and multivitamins but he was not accept the modified diet. On day 5 of hospitalization, a blood test showed a moderate increase in selenium, so from day10 of hospitalization, sodium selenate administration was increased to 80 µg/d. On day 12 of hospitalization, he was discharged due to elevation of selenium and vitamin C levels. At the time of discharge, perioral erosions and gingival swelling/bleeding had improved. He was able to take 1 g/d of multivitamin medicine including 2500 units retinol, 1 mg thiamine nitrate, 1.5 mg riboflavin, 5 mg calcium pantothenate, 1 mg pyridoxine hydrochloride, 1μg cyanocobalamin, 37.5 mg ascorbic acid, 200 units ergocalciferol, 1 mg tocopherol, 10 mg nicotinamide, and 0.5 mg folic acid. Since he was not able to take selenium medicine, he was admitted to hospital to replenish selenium by intravenous infusion every 3 months. No symptoms of selenium deficiency or scurvy were observed 8 months from discharge.

The selenium level for specific snacks and lacto-fermenting drinks were measured by nductively coupled plasma mass spectrometry (ICP-MS) at Japan functional food analysis and research center. These selenium levels were below 0.1 µg/g.

## Discussion

This case was reported to suffer both selenium deficiency and scurvy from picky eating in 7-year-old boy with ASD.

There are some reports in the US, UK and Japan about the frequency of trace elements/vitamin deficiency due to picky eating in children with ASD [11–13]. The most common trace element/vitamin deficiency reported in children with ASD in Japan was vitamin A [11]. A decrease in a retinol protein was also observed in this Japanese patient. Regarding element/vitamin deficiency in children with ASD, lower vitamin D and calcium levels were often reported in the US. [12, 14], and low vitamin C and D levels were reported in the UK [13]. The reason for the differences in levels of trace elements/vitamins between countries may be due to differences in dietary preferences of children with ASD across counties. In this patient, vitamins A and C were low, but vitamin D was within normal levels.

Selenium deficiency is sometimes observed in cases of prolonged management of selenium-free enteral nutrition and high-calorie infusions, but selenium deficiency is unlikely to occur in normal life since selenium is abundant in seafood, meat, eggs, and cereals. The amount of selenium in different foods depends on the region and country due to selenium transferring from soil to crops [15, 16]. Except for a situation of a selenium free diet in Japan, no selenium deficiency was reported. We thought that macrocytic anemia were due to selenium deficiency and/or folic acid deficiency.

The recommended selenium intake for 7-year-old children is 30 µg/d [17]. This patient routinely had 2 L/d of the lacto-fermenting drink with the snacks. We estimated how much selenium could be included in the snack. Selenium comprises up to 70 ng/1 g weight of raw potatoes [18]. Considering that the snacks had a mass weight of 57 g/ box and that he consumed two boxes/d, it is calculated that the snacks contained only 8 μg of selenium, even if all of the snacks were composed of potato. In addition, according to the product information, this snack included frying during the cooking process, which is assumed to have further reduced the amount of selenium [19]. The amount of selenium intake from the snacks was also considered up to only 0.6–5.6 µg/d in this patient [20]. The amount of selenium in lacto-fermenting drink was unknown. The Japanese-language literature described that the selenium content of lacto-fermenting drink was 0.00 µg/g wet weight [21]. The selenium level for specific snacks and lacto-fermenting drinks were below 0.1 µg/g in our research. This case was overwhelmingly deficient in selenium intake.

Under imbalanced diet conditions, scurvy is more frequent than selenium deficiency. Since vitamin C deficiency secondary to either selective eating or parents limiting a child’s diet may not be a common occurrence, it may also not be considered rare, with 61 published studies in the last 18 years [20]. The recommended vitamin C dose is 30 mg/d for a 7-year-old [21], which should easily be achievable. The snack was made of fried potato. Potatoes contain 43.2 mg vitamin C/100 g [22], but he had little or no vitamin C intake, as vitamin C is water-soluble, and rinsing and other processing may have led to the onset of scurvy. Vitamin C is abundant in vegetables, fruits, and potatoes, but it has the lowest residual rate among all vitamins after cooking. Reported differences in vitamin residuals between uncooked and cooked foods show that vitamin content decreases with frying and boiling [23].

The patient also had decreased vitamin B1, folic acid, and retinol protein. Vitamin B1 deficiency causes psychiatric and neurological symptoms such as depression, as well as motor/digestive symptoms and Wernicke-Korsakoff syndrome. Folic acid deficiency causes anemia, fatigue, paleness, irritability, shortness of breath, and in severe cases, tongue pain, diarrhea, decreased sense of taste, depression, confusion, and dementia. Retinol protein is closely related to vitamin A levels, and vitamin A deficiency can present with visual disturbances such as night blindness and photophobia. The diagnosis of mental retardation was made prior to the onset of the picky eating, and therefore, it was determined that the neurological symptoms were not related to low vitamin B1. No other symptoms that could be seen with vitamin B1 deficiency, folic acid deficiency, or retinol protein deficiency were observed.

Although there are few reports in Google Scholar or PubMed of scurvy and selenium deficiency due to an imbalanced diet, there are many reports of trace element deficiencies or vitamin deficiencies, including selenium, due to picky eating. We were unable to confirm whether there were really no cases of both scurvy and selenium deficiency by scrutinizing each of those cases.

## Conclusion

We reported a complicated case of both selenium deficiency and scurvy due to an imbalanced diet of snacks and lacto-fermenting drinks in a 7-year-old boy with autism spectrum disorder. Extremely picky eating induced both selenium deficiency and scurvy simultaneously. In patients with imbalanced diets, regular blood tests including trace elements and vitamins are considered necessary.

## Data Availability

Not applicable.

## Abbreviations

ASD: Autistic spectrum disorder
ADHD: Attention deficit hyperactivity disorder
D: Day
dL: Deciliter
DQ: Developmental quotient
DSM: Diagnostic and Statistical Manual of Mental Disorders
g: Gram
Kg: Kilogram
L: Liter
µg: Microgram
mg: Milligram
NADH: Nicotinamide-adenine dinucleotide reduced form
NF-κB: Nuclear factor-kappa B
PPAR: Peroxisome proliferator-activated receptor
SD: Standard deviation

## References

[1] Gandhi UH, Kaushal N, Ravindra KC, Hegde S, Nelson SM, Narayan V (2011). Selenoprotein-dependent up-regulation of hematopoietic prostaglandin D2 synthase in macrophages is mediated through the activation of peroxisome proliferator-activated receptor (PPAR) gamma. J Biol Chem..

[2] Etani Y, Nishimoto Y, Kawamoto K, Yamada H, Shouji Y, Kawahara H (2014). Selenium deficiency in children and adolescents nourished by parenteral nutrition and/or selenium-deficient enteral formula. J Trace Elem Med Biol..

[3] van Rij AM, Thomson CD, McKenzie JM, Robinson MF (1979). Selenium deficiency in total parenteral nutrition. Am J Clin Nutr..

[4] D’Haese PC, De Broe ME (1996). Adequacy of dialysis: Trace elements in dialysis fluids. Nephrol Dial Transplant.

[5] Strumila R, Lengvenyte A, Olie E, Seneque M, Dupuis-Maurin K, Alacreu-Crespo A (2022). Selenium deficiency is associated with disease severity, disrupted reward processing, and increased suicide risk in patients with Anorexia Nervosa. Psychoneuroendocrinology..

[6] Ryohei K, Mari H, Chiharu K (2021). Thyoid function in patients with selenium deficiency exhibits high free T4 toT3 ratio. Clin Pediatric Endocrinopathy.

[7] Abdullah M, Jamil RT, Attia FN. Vitamin C (ascorbic acid). StatPearls [Internet]. StatPearls Publishing; 2022. 29763052

[8] Robert S, Thomas U (2001). Scurvy identified in the emergency department: a case report. J Emerg Med.

[9] May JM, Cobb CE, Mendiratta S, Hill KE, Burk RF. Reduction of the ascorbyl free radical to ascorbate by thioredoxin reductase. J Biol Chem. 1998;273:23039–45. 10.1074/jbc.273.36.23039 9722529

[10] Ma NS, Thompson C, Weston S (2016). Brief Report: Scurvy as a manifestation of food selectivity in children with autism. J Autism Dev Disord..

[11] Tanoue K, Minami T, Syou N, Fujita J, Toyohara K, Kato H (2017). Food repertoire and nutritional deficiency in Japanese children with autism spectrum disorders. Japanese J Child Adolescent Psychiatry (in Japanese)..

[12] Bandini LG, Anderson SE, Curtin C, Cermak S, Evans EW, Scampini R (2010). Food selectivity in children with autism spectrum disorders and typically developing children. J Pediatr..

[13] Emond A, Emmett P, Steer C, Golding J (2010). Feeding symptoms, dietary patterns, and growth in young children with autism spectrum disorders. Pediatrics..

[14] Zimmer MH, Hart LC, Manning-Courtney P, Murray DS, Bing NM, Summer S (2012). Food variety as a predictor of nutritional status among children with autism. J Autism Dev Disord..

[15] Hurst R, Siyame EW, Young SD, Chilimba AD, Joy EJ, Black CR (2013). Soil-type influences human selenium status and underlies widespread selenium deficiency risks in Malawi. Sci Rep..

[16] Gao J, Liu Y, Huang Y, Lin Z, Bañuelos GS, Lam MH (2011). Daily selenium intake in a moderate selenium deficiency area of Suzhou. China. Food Chem..

[17] Kipp AP, Strohm D, Brigelius-Flohé R, Schomburg L, Bechthold A, Leschik-Bonnet E (2015). Revised reference values for selenium intake. J Trace Elem Med Biol..

[18] Navarro-Alarcon M, Cabrera-Vique C (2008). Selenium in food and the human body: A review. Sci Total Environ..

[19] Dong Z, Liu Y, Dong G, Wu H (2021). Effect of boiling and frying on the selenium content, speciation, and in vitro bioaccessibility of selenium-biofortified potato (Solanum tuberosum L.). Food Chem..

[20] US Department of Agriculture, Agricultural Research Service. USDA national nutrient database for standard reference, release 28. Nutrient data laboratory home page. 2011.

[21] Yamamoto R, Okita R, Hiruta A, Konno T. Selenium in foodstuff (in Japanese). Res Rep Shokei Gakuin College. 2006;52:205–18.

[22] Hahn T, Adams W, Williams K (2019). Is vitamin C enough? A case report of scurvy in a five-year-old girl and review of the literature. BMC Pediatr..

[23] German Nutrition Society (DGE) (2015). New Reference Values for vitamin C intake. Ann Nutr Metab..

[24] Hamouz K, Lachman J, Pivec V. Influence of environmental conditions and way of cultivation on the polyphenol and ascorbic acid content in potato tubers, 45. Food and Agriculture Organization of the United Nations. 1999;45:293–8.

[25] Ikanone CEO, Oyekan PO (2014). Effect of Boiling and Frying on the Total carbohydrate, vitamin C and Mineral Contents of Irish (Solanun tuberosum) and Sweet (Ipomea batatas) Potato tubers. Nigerian Food J.

